# The Effects of Serotonin Receptor Type 7 Modulation on Bowel Sensitivity and Smooth Muscle Tone in Patients With Irritable Bowel Syndrome

**DOI:** 10.7759/cureus.42532

**Published:** 2023-07-27

**Authors:** Usama Osman, Abishek Latha Kumar, Aishwarya Sadagopan, Anas Mahmoud, Maha Begg, Mawada Tarhuni, Monique N. Fotso, Natalie A Gonzalez, Raghavendra R Sanivarapu

**Affiliations:** 1 Internal Medicine, California Institute of Behavioral Neurosciences & Psychology, Fairfield, USA; 2 Internal Medicine, Pediatrics, California Institute of Behavioral Neurosciences & Psychology, Fairfield, USA; 3 Obstetrics and Gynecology, California Institute of Behavioral Neurosciences & Psychology, Fairfield, USA; 4 Pediatrics, California Institute of Behavioral Neurosciences & Psychology, Fairfield, USA; 5 Pulmonary and Critical Care Medicine, California Institute of Behavioral Neurosciences & Psychology, Fairfield, USA

**Keywords:** 5-ht7, visceral hyperalgesia, visceral hypersensitivity, bowel sensitivity, serotonin receptor type 7, ibs

## Abstract

Irritable bowel syndrome (IBS) is a gut-brain disorder involving alterations in intestinal sensitivity and motility. Currently, IBS has no cure, and treatment focuses on the management of symptoms. The diverse, and sometimes contradictory, collections of symptoms reported across the different subtypes of IBS make treatment, as well as diagnosis, difficult for physicians. All subtypes of IBS have one symptom in common: abdominal pain caused by differences in the level of visceral sensitivity. Though current research on this topic is in its infancy, some researchers have proven, through experimental studies, that 5-hydroxytryptamine (serotonin) receptor type 7 (5-HT7) affects both visceral sensitivity and smooth muscle tone in the bowel. Therefore, this review will be discussing the future possibility of alleviating abdominal pain in patients with IBS and related disorders by modulating the 5-HT7 receptor.

## Introduction and background

Irritable bowel syndrome (IBS), a gut-brain interaction disorder, is one of the most pervasive functional gastrointestinal disorders that negatively affects a patient’s quality of life. IBS patients struggle with symptoms that affect their ability to live their day-to-day lives. Without a cure, treatment focuses on the management of individualized symptoms, unfortunately without a high rate of success [1]. The prevalence of the disease varies from one country to another depending on the local diagnostic criteria. IBS symptoms and clinical presentation can differ from patient to patient, depending on their individual bowel sensitivity. Abdominal pain is the common thread between all patients as it relates to bowel sensitivity. Though research is in its infancy, it may be possible to alleviate abdominal pain for patients with IBS and related disorders by elevating the pain threshold through 5-hydroxytryptamine (serotonin) receptor type 7 (5-HT7) receptor modulation. This review will summarize and discuss the current research and discoveries on this topic, as well as explore the connection between abdominal pain in IBS and serotonin receptor type 7. 

Methods

The scale for the assessment of narrative review articles (SANRA) was used to assess the quality of articles selected for review. All research sources were collected from PubMed, Pubmed Central, and Google Scholar databases to explore articles related to serotonin receptors and IBS. The Food and Drug Administration (FDA) website was also searched for any new updates regarding IBS treatments. As an example of how the search strategy was structured, the following search strategies were used to locate articles on Pubmed. A keyword search for “IBS,” “serotonin receptor 7,” “IBS AND serotonin receptor 7,” “5-HT7,” "bowel sensitivity," and "visceral hypersensitivity" was made, and then the results were meshed with “biosynthesis,” “adverse effects,” “immunology,” and “therapeutic use.” Specifically, studies to be included were regarding visceral hyperalgesia and bowel sensitivity related to IBS, serotonin pathways, and their relation to IBS, as well as research on serotonin type 7 and its relation to bowel function. Article types of particular interest were randomized controlled trials (RCTs), systematic reviews, observational studies, and meta-analyses on the relationship between serotonin receptor type 7 and IBS. This cross-reference between 5-HT7 and IBS produced few results. Additionally, only articles published in English or studies available with English translations were included. In exploring the existing documentation of articles, there were a large number of articles concerning IBS and serotonin receptors independently. To narrow this wide field of several hundred articles, the following exclusion criteria were also applied: studies that did not evaluate serotonin receptor modulation specifically and studies that did not include the relevant link between serotonin and an associated IBS response. The results of this search included reviews of current and past literature, animal studies, and observational studies. 

## Review

Irritable bowel syndrome

IBS is the most commonly diagnosed functional gastrointestinal disorder worldwide. Symptoms of IBS include differences in bowel routines and consistency, increased gas in the intestinal tract, and increased abdominal pain [2]. The severity and duration of IBS symptoms can vary significantly from person to person, generally lasting between two and five days per episode. Currently, there is no cure for IBS. Historically, treatments have focused on symptom management, unfortunately with limited success [1].

Due to an absence of clear pathological markers, as found in bowel inflammatory diseases, the diagnosis of IBS is made based on the presence of symptoms and further classified by the nature of the predominant stool pattern: IBS-C (constipation), IBS-D (diarrhea), IBS-M (mixed or alternating), and IBS-U (un-subtyped, in which the stool consistency does not meet the criteria for IBS-C, D, or M) [2,3]. It is often difficult to directly diagnose IBS in patients, as its individual symptoms overlap with other gastrointestinal conditions. Symptoms of IBS have a low level of specificity when viewed independently, so an effective diagnosis has been developed on several symptoms that often occur in tandem [1].

Diagnosis

The first comprehensive diagnostic criteria for IBS was developed in 1978 and named the Manning criteria after its publisher, which has since been succeeded by the Rome criterion: Rome I, II, III, and, most recently, the updated Rome IV criteria [1]. The principal features of an IBS diagnosis consist of abdominal pain from bowel sensitivity and bowel discomforts, such as bloating, diarrhea, or constipation. Bowel sensitivity is related to different factors, such as chemicals in food, mechanical stress due to bowel impaction, the amount of gas in the intestinal tract, and psychological stress. A clinical history of some combination of these symptoms must occur more than three days per month over a three-month period preceding diagnosis, and the onset of these symptoms must be more than six months prior to diagnosis, per the Rome IV criteria [1].

In addition to the Rome IV diagnostic criteria, there are supporting symptoms that are often related to IBS but not required for clinical diagnosis. An analysis of the stool consistency and bowel movement frequency can be used to distinguish diarrhea-type IBS from other gastrointestinal diseases. Symptoms such as unpredictable bowel movements and extreme frequencies in excess of three times per day or less than three times per week are noted frequently in IBS patients. Additional symptoms of straining or mucus in the stool may also support an IBS diagnosis. IBS-linked symptoms extend beyond digestive issues as well: “chronic fatigue, fibromyalgia, uro-gynecological symptoms, muscle and joint pain, sleep disorder, and psychological co-morbidity (such as anxiety and depression)” [4] are also cited as common co-occurrences. On the topic of co-morbidity, IBS patients have an increased prevalence of traumatic life events during childhood, such as abuse, and the degree of trauma is often correlated to the severity of the IBS [5]. The difficulty in diagnosing IBS is the frequent overlap of symptoms with other gastrointestinal disorders and the lack of direct testability [6]. Certain symptoms, such as “fever, gastrointestinal bleeding, anemia, weight loss, abdominal mass, nocturnal symptoms, fecal soiling or family history of colon cancer,” [6] are indicative of a more serious diagnosis and require additional testing. The crux of IBS is the current lack of successful medical treatment options to normalize bowel sensitivity, which may underscore all IBS subtypes [6].

IBS Treatment Options

There is no cure for IBS; restoring the digestive process to its healthy, normal level of functioning is not currently possible for patients with IBS. One of the greatest hurdles for patients with IBS is struggling to be accurately diagnosed. Diagnosis is achieved through a process of elimination through gastroenterological testing to rule out more severe diseases, such as colon cancer [1]. There are three mainstream avenues of treatment for managing the symptoms of IBS, which consist of dietary interventions, pharmacological medications, and psychotherapy, but there is no silver bullet. Treatment must be individualized to the specific symptom patterns of each IBS patient [1].

The first recommended course of treatment is dietary intervention [7]. Food-related intolerances after ingestion are a frequently reported symptom of IBS patients and are often noticeably worse after the ingestion of certain foods. This is a physiological reaction and not an immune response. The difference between intolerance and allergy is clinically significant and makes identifying problem foods extremely difficult in the absence of a structured dietary intervention, such as the low fermentable oligosaccharides, disaccharides, monosaccharides, and polyol (FODMAP) diet or a systematic and carefully documented elimination diet [1]. Manipulation of gut microbiota and herbal supplements may also ease symptoms [1]. 

If dietary intervention proves to be ineffective, pharmacological treatment is often recommended. As there is no single treatment for all IBS types, pharmacological treatment is tailored to managing patient symptoms as effectively as possible with minimal side effects. Antispasmodics are prescribed for abdominal pain, laxatives for patients with IBS-C, and antidiarrheals for patients with IBS-D [1]. Although a variety of pharmaceutical medications are available, they are not always effective and may cause intolerable side effects.

If the symptoms of IBS do not lessen under dietary and pharmacological treatment, psychotherapy is recommended. The biopsychosocial approach suggests that abdominal pain and other symptoms of IBS increase symptoms of anxiety and depression in patients and that the psychological symptoms affect motor function, sensory threshold, and stress reactivity in the gut [4]. Therapy aimed at decreasing psychological distress, hyper-vigilance, and catastrophic thinking may be helpful in improving peripheral regulation of gut function and brain-gut signaling [4].

Patient quality of life is greatly affected as symptoms of IBS, especially non-IBS-C types, may potentially cause feelings of shame, fearfulness, or embarrassment. Across studies of quality of life on the 36-Item Short Form Survey (SF-36), which measures quality of life, IBS patients reported lower scores than average and worse in several categories than patients with chronic kidney disease, diabetes mellitus, or gastroesophageal reflux disease. Quality of life was reported to be significantly improved when symptoms respond to intervention; therefore, successful treatment options for IBS are of the utmost importance [1].

Serotonin (5-HT)

The discovery of the neurotransmitter serotonin, in 1949 by Maurice Rapport, began a branch of research about this molecule [8,9,10,11]. Abbreviated as 5-HT, for the molecule 5-hydroxytryptamine, serotonin is “involved in numerous diseases of the (central nervous system) CNS (e.g., depression, anxiety, schizophrenia, obsessive-compulsive disorders, addiction, Parkinson’s disease) and peripheral organs (e.g., gastrointestinal disorders, cardiac arrhythmia, hypertension)" [12]. However, the impact of serotonin extends well beyond its association with functions in the brain [13]. Approximately 90% of the serotonin in humans is produced in the gastrointestinal system, for use in digestive processes [14,15,16]. 

What makes serotonin a particularly adaptable neurotransmitter is its variety of receptors. As serotonin research has advanced over time, focus has been made on the individual serotonin receptor subtypes and their associated biological processes [8]. “To date, seven distinct families of 5-HT receptors have been identified, with some families consisting of various subpopulations. Five of the seven known families (5-HT1, 5-HT2, 5-HT3, 5-HT4, and 5-HT7 receptors) are expressed in the gut, with the 5-HT3 and 5-HT4 subtypes being the most extensively studied" [17]. Though much research has been done, medications targeting 5-HT3 and 5-HT4 have been proven to be ineffective, due to intolerable side effects [1,18].

Depending on the symptoms and IBS subtype, it may be possible to successfully manage the symptoms of IBS by altering bowel sensitivity. The distinct effect that 5-HT7 modulation has on improving bowel sensitivity and inducing smooth muscle relaxation may potentially increase the pain threshold in the bowel, thus alleviating this symptom of IBS. Although more research is required, the surveyed literature does support the hypothesis that 5-HT7 potentially has therapeutic effects for patients with IBS. Antagonizing 5-HT7 may have therapeutic benefits in the acute phase, as well as long term, for IBS patients [19]. During the acute phase, it will lead to the relaxation of circular smooth muscles in the colon, which may then relieve the tension, due to gas and constipation in IBS-C. Antagonizing 5-HT7 may also decrease the frequency of bowel movements, thus improving diarrhea in patients with IBS-D. In the long run, inhibition of this receptor may also lower the rate of neurite growth and their length in the mucosal layer of the colon in IBS patients, potentially leading to a significant decrease in bowel sensitivity and abdominal pain [20]. 

It may be possible to successfully manage the symptoms of IBS by altering bowel sensitivity. Many unanswered questions remain, and numerous new avenues of research exist that warrant further investigation. As research about 5-HT7 continues, the potential benefits for patients with IBS and related disorders will become more clear.

5-HT7 Mechanism of Action

Serotonin's last receptor, 5-HT7, was discovered in 1993 [8]. It was found to function in the CNS as well as other peripheral organs. According to experimentation done circa 2012, 5-HT7 receptors were established to have roles in “circadian rhythm, thermoregulation, … and mood disorders including depression" [17]. Of interest to the topic of this review, receptors were also found to be active in the stomach, colon, and small intestine, with lower activity in the liver, kidney, and spleen [17]. 5-HT7 receptors were found on smooth muscle cells, enteric neurons, intestinal dendritic cells, and intestinal lymphoid tissue, all of which help regulate processes in the bowel system [17]. 

5-HT7 has been linked to controlling smooth muscle relaxation in the colon and other parts of the intestinal system, which are responsible for bowel motility [17]. Through experimentation, it was found that antagonizing 5-HT7 receptors resulted in the relaxation of smooth muscle in the intestines, thus identifying a positive correlation between 5-HT7 activity and bowel motility [14]. This effect would be most beneficial when applied to IBS-D patients to decrease bowel movements to a normal frequency [17].

The correlation between abdominal pain and visceral sensitivity

**Figure 1:**
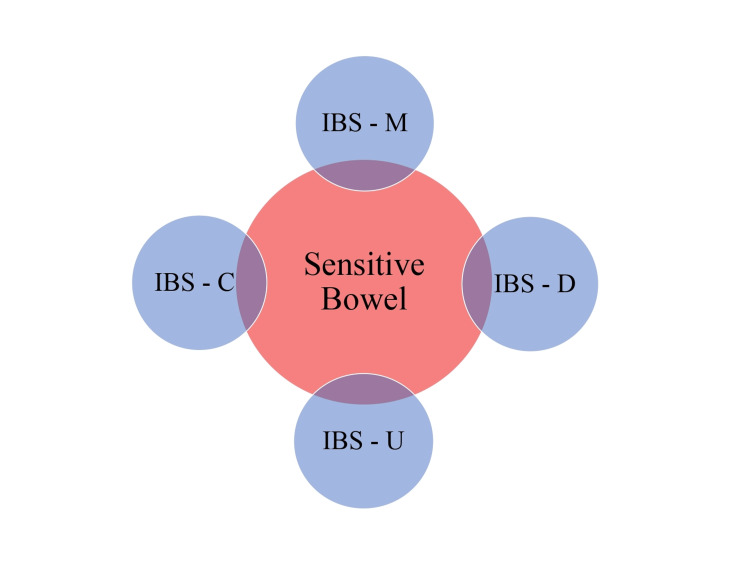
Involvement of Bowel Sensitivity in All IBS Types

Visceral sensitivity refers to the measure of how sensitive internal organs are to pain [21,22]. The digestive process should not be painful, so when a patient reports abdominal pain, it is a cause for concern [5]. Increased visceral sensitivity may explain the abdominal pain in IBS patients, as a result of mechanical pressure, chemical composition of ingested food, or psychological stress [23]. “Studies report that 35-90% of IBS patients demonstrate (increased) visceral (sensitivity),” which causes a lowered threshold for pain [5,24]. The neural pathways for the transmission of abdominal pain were found to be affected by 5-HT7 activity at the peripheral tissue level and at the CNS level [5,25]. Abdominal pain may be associated with dysfunctional pain transmission through these pathways [26]. Figure 1 illustrates the involvement of bowel sensitivity in all types of IBS.

To understand the role of 5-HT7 in pain modulation, it is necessary to establish how bowel pain is communicated to the brain. Afferent peripheral nerves in the bowel system are responsible for registering different types of stimuli, which are then communicated to the CNS [27]. The spinothalamic tract sends sensory stimuli to the thalamus, which is linked to sensory perception and cognitive and mood processes in the brain. The ipsilateral dorsal columns also transfer sensory information to the thalamus. The role of the CNS in this process is to actively filter stimuli from the afferent peripheral nerves by either inhibiting or facilitating signal transduction [26]. In patients with increased visceral sensitivity, there may be a discrepancy in this process at the afferent nerve level or at the CNS level, which may lead to an increased pain response [28].

Effects of 5-HT7 Modulation

The activity of afferent pain receptors has been shown to be linked to 5-HT7 receptors in animal studies [27,29]. The regulation of 5-HT7 receptor activity has been linked to afferent nociceptor responses after the injection of both 5-HT7 agonist and antagonist in separate trials. The injection of 5-HT7 agonist led to an increase in the c-Fos gene production in nociceptors, which marks recent neuron activity and therefore increased the severity of the pain stimuli. The injection of a 5-HT7 antagonist, followed by a 5-HT7 agonist, demonstrated a significantly inhibited afferent nociceptor response, indicating that increased 5-HT7 receptor activity is responsible for higher pain responses in subjects [30].

The biomechanical reason for this increased pain response lies in how the activity in the 5-HT7 receptor affects nerve length in the mucosal layer of the bowel [31,32]. “Mucosal neurite outgrowth contributed to intestinal hypernociception … A positive-feedback loop driving nerve fiber elongation was observed between serotonin and neurotrophins (showing that) 5-HT7 plays a key role in (the mucosal neurite outgrowth)" [14]. There is a connection between the intensified mucosal layer innervation and visceral sensitivity [14,33]. “An aggravating loop between serotonin and neurotrophin was identified in this study for intensifying mucosal innervation and intestinal nociception" [14]. This neurite outgrowth may cause a lowering of the pain threshold, which affects visceral sensitivity in IBS patients. 

Another bowel condition that is related to 5-HT7 is colitis: inflammation of the bowels. Although colitis is not an associated symptom of IBS, colitis demonstrates a link between bowel inflammation and 5-HT7 receptor activity. In considering the effect of 5-HT7 agonization and antagonization on induced colitis, it was found that inhibition by blockage or genetic ablation of 5-HT7 receptors increased the severity of colitis symptoms [34]. Some chemical methods for inducing colitis were linked to 5-HT7 receptors, and some were not, suggesting that the 5-HT7 colitis relationship is model specific. In dextran sulfate sodium (DSS)- and dinitrobenzene sulfonic acid (DNBS)-induced colitis, colitis severity was impacted by 5-HT7 modulation. While 5-HT7 antagonization yielded an increased severity of symptoms, 5-HT7 agonization led to significantly lowered colitis symptoms, thus proving that 5-HT7 has an anti-inflammatory effect on the bowel system for some inflammatory diseases [33]. It is also significant to note that it was found that, in IBS with diarrhea, the plasma level of serotonin increased, whereas, in patients with constipation, it decreased [35]. This is important, as it proves that serotonin plays different roles in different parts of the gastrointestinal system. 

## Conclusions

Depending on the symptoms and IBS subtype, it may be possible to successfully manage the symptoms of IBS by altering bowel sensitivity. The effects of 5-HT7 modulation affect bowel sensitivity and smooth muscle tone, thereby increasing the pain threshold in the bowel. This is a relatively recent topic in medical research, so information on increasing pain tolerance (ascending methods of limits) by antagonizing 5-HT7 is limited. Modulating this receptor may affect both motility and sensitivity of the bowel, potentially allowing IBS patients to improve their quality of life. Due to limited research on 5-HT receptors in the intestinal system, scientists do not yet fully understand the mechanisms of serotonin receptor modulation. Therefore, more research is needed. Prior research has proven that the 5-HT7 receptor, in particular, plays a role in intestinal functions, pathologies, and pathophysiology. Due to its potential to alleviate symptoms of IBS, research on this receptor is necessary to facilitate the advent of novel treatments for patients diagnosed with IBS and related disorders. 
